# Overexpression of a Minimal Domain of Calpastatin Suppresses IL-6 Production and Th17 Development via Reduced NF-κB and Increased STAT5 Signals

**DOI:** 10.1371/journal.pone.0027020

**Published:** 2011-10-27

**Authors:** Mikiko Iguchi-Hashimoto, Takashi Usui, Hajime Yoshifuji, Masakazu Shimizu, Shio Kobayashi, Yoshinaga Ito, Kosaku Murakami, Aoi Shiomi, Naoichiro Yukawa, Daisuke Kawabata, Takaki Nojima, Koichiro Ohmura, Takao Fujii, Tsuneyo Mimori

**Affiliations:** 1 Department of Rheumatology and Clinical Immunology, Graduate School of Medicine, Kyoto University, Kyoto, Japan; 2 Center for Innovation in Immunoregulative Technology and Therapeutics, Graduate School of Medicine, Kyoto University, Kyoto, Japan; French National Centre for Scientific Research, France

## Abstract

Calpain, a calcium-dependent cysteine protease, is reportedly involved in the pathophysiology of autoimmune diseases such as rheumatoid arthritis (RA). In addition, autoantibodies against calpastatin, a natural and specific inhibitor of calpain, are widely observed in RA. We previously reported that E-64-d, a membrane-permeable cysteine protease inhibitor, is effective in treating experimental arthritis. However, the exact role of the calpastatin-calpain balance in primary inflammatory cells remains unclear. Here we investigated the effect of calpain-specific inhibition by overexpressing a minimal functional domain of calpastatin in primary helper T (Th) cells, primary fibroblasts from RA patients, and fibroblast cell lines. We found that the calpastatin-calpain balance varied during Th1, Th2, and Th17 development, and that overexpression of a minimal domain of calpastatin (by retroviral gene transduction) or the inhibition of calpain by E-64-d suppressed the production of IL-6 and IL-17 by Th cells and the production of IL-6 by fibroblasts. These suppressions were associated with reductions in RORγt expression and STAT3 phosphorylation. Furthermore, inhibiting calpain by silencing its small regulatory subunit (CPNS) suppressed Th17 development. We also confirmed that overexpressing a minimal domain of calpastatin suppressed IL-6 by reducing NF-κB signaling via the stabilization of IκBα, without affecting the upstream signal. Moreover, our findings indicated that calpastatin overexpression suppressed IL-17 production by Th cells by up-regulating the STAT5 signal. Finally, overexpression of a minimal domain of calpastatin suppressed IL-6 production efficiently in primary fibroblasts derived from the RA synovium. These findings suggest that inhibiting calpain by overexpressing a minimal domain of calpastatin could coordinately suppress proinflammatory activities, not only those of Th cells but also of synovial fibroblasts. Thus, this strategy may prove viable as a candidate treatment for inflammatory diseases such as RA.

## Introduction

Rheumatoid arthritis (RA) is a chronic inflammatory disease characterized by symmetric polyarthritis, accompanied by joint destruction, and is widely regarded as a systemic autoimmune disease. Although the pathogenesis of RA is not yet fully understood, several studies indicate that helper T (Th) cells assume an important role. Naïve CD4^+^ T cells differentiate into distinct types of Th cells: IFN-γ-producing Th1 cells, IL-4-producing Th2 cells, and the recently defined IL-17-producing Th17 cells [1], [2], [3]. IL-12, STAT4, and T-cell-specific T-box transcription factor (T-bet) signals are known to be important for Th1 development, as are IL-4, STAT6, and GATA binding protein 3 (GATA-3) signals for Th2 development [4], [5], [6], and IL-1β, TGF-β, IL-6, IL-23, STAT3, and retinoic acid-related orphan receptor gamma t (RORγt) signals for Th17 development [1], [2], [7], [8], [9], [10], [11]. Although Th1 cells were formerly considered the main effector cells for the pathogenesis of autoimmune arthritis, we now know that Th17 cells play an essential role in autoimmune arthritis in mice. For example, in collagen-induced arthritis (CIA) model mice, systemic or local IL-17 gene transfer aggravates CIA and enhances joint destruction, whereas the administration of an IL-17-blocking antibody ameliorates CIA, even after disease onset [12], [13]. However, whether this finding can be applied to human RA remains controversial. Two independent groups including ours have reported that Th1 cells are dominant while Th17 cells are scarce in the synovial tissues and fluids of RA patients [14], [15].

On the other hand, various autoantibodies such as rheumatoid factor (RF), anti- citrullinated protein [16], anti-type II collagen (CII) [17], and anti-glucose-6-phosphate isomerase (GPI) antibody [18] have been proposed to have pathogenic roles in RA. In particular, we and others have documented the presence of anti-calpastatin (a natural specific inhibitor of calpain) antibodies in RA [19], [20] and psoriasis [21]. The sensitivity and specificity of anti-calpastatin antibodies for diagnosing RA are 83% and 96%, respectively [22]. Positivity for these antibodies correlates with serological markers of the disease activity [23], [24], and their detection is applicable to the diagnosis of early RA [25]. Furthermore, these anti-calpastatin antibodies are known to inhibit the function of calpastatin [19], [21]. Calpain, a calcium-dependent cysteine protease, is thought to modulate various intracellular signaling pathways [26], and may contribute to the pathogenesis of RA. For example, calpain is reportedly up-regulated in the synovial tissue of RA and CIA mice [27], [28], and has been shown to degrade the matrix component of articular cartilage [29]. One report demonstrated the successful treatment of CIA with calpain inhibitor I [30]. There are reports that calpain is involved in LFA-1-mediated T-cell adhesion [31], as well as T-cell proliferation via α-actinin-modification [32]. Thus, it is conceivable that excess calpain in the joints of RA patients whose calpastatin activity is inhibited by the presence of anti-calpastatin antibodies could contribute to the pathophysiology of RA. In this regard, we also reported that E-64-d, a membrane-permeable calpain inhibitor, ameliorates anti-collagen antibody-induced arthritis (CAIA), another animal model of RA [33]. Taken together, these results suggest that an insufficiency of calpastatin or an overabundance of calpain contributes to the pathogenesis of inflammatory diseases such as RA.

In this study, we found that the calpastatin-calpain balance modulated the fate of Th-cell development, and that inhibiting calpain by overexpressing a minimal functional domain of calpastatin suppressed IL-6 production and Th17 development in primary Th cells, and the production of IL-6 by primary human fibroblasts from the RA synovium. We also examined the mechanisms underlying these effects.

## Materials and Methods

### Mice and reagents

BALB/c mice (6–10-weeks old) were purchased from Charles River Laboratories. The mice were maintained in our animal facility under specific pathogen-free conditions and treated in accordance with the guidelines for animal care at Kyoto University. All animal procedures were approved by the ethics committees of Kyoto University (MedKyo 10142). Calpastatin cDNA was obtained from a λgt11 cDNA library [19]. Calpain cDNA was purchased from Open Biosystems, and E-64-d was from Peptide Institute (Japan). Recombinant human IL-2, and murine IL-4, IL-6, and IL-12 were purchased from PeproTech Inc.; human TGF-β was from R&D Systems. Monoclonal antibodies to murine CD3 (2C11) and CD28 (37.51) were purchased from eBioscience and BD Biosciences, respectively. Anti-IL-4 antibody (11B11) was purchased from BioLegend Inc., and anti-IFN-γ antibody (XMG1.2) from BD Biosciences. Phoenix 293T cells and NIH-3T3 cells (ATCC) were maintained in DMEM with 10% FCS, 10 U/ml penicillin, 10 µg/ml streptomycin, 50 µM 2-ME, and 20 mM HEPES. CD4^+^ T cells from mice were maintained in RPMI1640 using the supplements listed above. All other reagents, unless stated otherwise, were purchased from Invitrogen Corp.

### Cells and cultures

Naïve CD4^+^ T cells derived from murine splenocytes were purified by negative selection using the Mouse CD4^+^ T cell Isolation Kit, followed by positive selection using anti-mouse CD62L MicroBeads (Miltenyi Biotec). Naïve CD4^+^ T cells were stimulated with 4 µg/ml plate-bound anti-CD3 monoclonal antibodies and 2 µg/ml soluble anti-CD28 monoclonal antibodies (eBioscience) under neutral (no cytokine) conditions, or Th1 (IL-12, 5 ng/ml)- or Th2 (IL-4, 20 ng/ml)-promoting conditions, then expanded with 10 U/ml human IL-2. For the Th17-promoting condition, naïve CD4^+^ T cells were stimulated with 1 µg/ml plate-bound anti-CD3 monoclonal antibodies and 0.5 µg/ml soluble anti-CD28 monoclonal antibodies in the presence of 20 ng/ml IL-6, 5 ng/ml TGF-β, and 20 ng/ml anti-IFN-γ antibody.

Human synovial tissues were obtained from RA patients undergoing joint replacement or the subcutaneous puncture of knee joints. Synovial tissue was dissected into small pieces with scissors, and digested with Liberase TM Research Grade (Roche Diagnostics). Synovial cells were then seeded into dishes, and infected with retrovirus several days later. All human materials were used under the approval of the Ethics Committee of Kyoto University, and written informed consent was obtained from all patients (E458).

### Plasmid construction and retroviral transduction

The retrovirus vector (pBMN-IRES-EGFP) was kindly provided by Dr. G. Nolan (Stanford University). The cDNAs for full-length calpastatin or calpain were inserted into the pBMN-IRES-EGFP vector using the EcoRI and EcoRI/NotI sites, respectively. The minimal functional domains of calpastatin were cloned by PCR using the following primers: sense primer 5′-GAA TTC AGA TCT GCC ACC ATG GAT GCT GCT TTG GAT-3′, for domains IABC and IAB, anti-sense primer, 5′-GAA TTC GCG GCC GCC TAG GTG AAG TCA GAT GAC AA-3′, for domain IABC, 5′-GAA TTC GCG GCC GCC TAG GGT TTA GCC AAT AGT TC-3′ for domain IAB, sense primer 5′-GAA TTC AGA TCT GCC ACC ATG GAC CTC GAT GAT GCC TTG-3′ for domain IVABC and IVAB and anti-sense primer 5′- GAA TTC GCG GCC GCC TAC AGA TCT CCT GAG AGA GCA TC-3′ for domain IVABC, 5′- GAA TTC GCG GCC GCC TAA TTA TCA TCC AGG AGA TG-3′ for domain IVAB. The PCR products were cloned into a pCR4 Blunt-TOPO vector (Invitrogen) followed by bidirectional sequencing, then subcloned into the pBMN-IRES-EGFP vector using the EcoRI and NotI sites. Retroviral transfection and infections were performed as previously described [5]. Naïve CD4^+^ T cells or NIH-3T3 cells were infected with collected viral supernatants containing 2 µg/ml polybrene (Sigma-Aldrich).

### PKH-26 labeling and ATP measurement to assay cell proliferation

To analyze the effect of retrovirus infection on the proliferation of Th cells, Th cells were stained with the dye PKH-26 (Sigma-Aldrich, St. Louis, MO), according to the manufacturer's instructions, then stimulated with anti-CD3/28 monoclonal antibodies. The fluorescence intensities of the infected cells were then analyzed by FACS. To analyze the effect of retroviral infection on the proliferation of primary fibroblasts from RA patients, the GFP-positive cells were sorted and plated into 96-well plates at 1000 cells/well. Forty-eight and 72 hours after plating, ATP was measured with a CellTiter-Glo® Assay kit according to the manufacturer's instructions (Promega Corporation).

### Calpain activity assay and ELISAs

Calpain activity was assayed as previously described [19]. ELISAs for IL-6 and IL-17 using Ready-set-Go! (eBioscience), according to the manufacturers' protocols.

### Intracellular cytokine and IκBα staining

Intracellular cytokine staining was performed as previously described [5]. APC-conjugated anti-mouse IFN-γ (XMG1.2), PE-conjugated anti-mouse IL-4 (11B11, eBioscience), anti-mouse IL-6 (MP5-20F3), and anti-mouse IL-17 (TC11-18H10) were used to detect these cytokines in cells. As negative controls, APC- or PE-conjugated isotype-matched monoclonal antibodies were used. Intracellular staining of IκBα was performed using an anti-IκBα antibody (sc-847) and APC-conjugated goat anti-rabbit IgG F(ab)^2^ antibody (sc-3846), both from Santa Cruz Biotechnology, according to the recommended protocol of Santa Cruz Biotechnology. The analysis was performed on a FACS Calibur with Cell Quest software (BD Biosciences). All reagents were purchased from BD Biosciences unless otherwise stated.

### Western blotting and densitometry

Protein extraction and Western blot analysis were performed by standard methods as previously described [34]. Anti-μ-calpain large subunit specific antibody (#2556), anti-phosphorylated STAT5 antibody (#9359), and the NF-κB Pathway Sampler Kit (#9936) were purchased from Cell Signaling Technology. Anti-calpastatin antibody (sc-20779), anti-actin antibody (sc-1616), anti-phosphorylated STAT3 antibody (sc-8059), anti-STAT3 antibody (sc-482), anti-STAT5 antibody (sc-835), and anti-NF-kB p65 antibody (sc-109) were purchased from Santa Cruz Biotechnology. Anti-calpain small subunit (CPNS) antibody (826-M01) was obtained from Abnova Corp. To analyze phosphorylated STAT3 and STAT5 in Th cells, the cells were incubated with X-VIVO™ 20 (without FCS, Lonza Walkersville, Inc.) for the last six hours and then lysed. After SDS-PAGE and electrotransfer to a membrane, the blotted membranes were blocked with TBS containing 5% non-fat milk and 0.05% Tween-20, probed with the indicated antibody, and developed using the Supersignal Chemiluminescence kit (Pierce Chemical Co.). The chemiluminescence signals were detected with BioMax Light film (Kodak) or an ImageQuant LAS 4000 mini (GE Healthcare). After stripping, the membranes were reprobed. The band densities on western blots were scanned and measured using NIH Image.

### Electrophoresis mobility shift assay

Nuclear extracts were prepared using NE-PER nuclear and cytoplasmic extraction reagents (Thermo Fisher Scientific Inc.). Electrophoresis mobility shift assays (EMSAs) were performed by incubating nuclear extracts and biotin-labeled double strand probes in a binding buffer (10 mM Tris, 50 mM KCl, 1 mM DTT, pH 7.5) at room temperature for 20 min. For the NF-κB-binding motif, the sequences of the oligonucleotides used for EMSA were: sense, 5-biotin/AGT TGA GGG GAC TTT CCC AGG-3; and anti-sense, 5-biotin/CCT GGG AAA GTC CCC TCA ACT-3 (Invitrogen). Probes labeled at the 5′ end with biotin and non-labeled probes were both prepared by annealing these two oligonucleotides. For super-shift experiments, the binding reactions were conducted in the presence of specific antibodies to p65 protein (sc-109, Santa Cruz). The protein–DNA complexes were subjected to electrophoresis on 5% polyacrylamide gels in 0.5×TBE and transferred onto a positive-charged nylon membrane (Hybond N+, GE Healthcare). The UV cross-linked membrane was then blocked and incubated with horseradish peroxidase-conjugated streptavidin, and visualized with a Lightshift chemiluminescent EMSA kit (Pierce Chemical Co.) using an ImageQuant LAS 4000 mini (GE Healthcare). Band densities were measured using NIH Image.

### RNA interference (RNAi)

Predesigned Stealth Select siRNAs against CPNS, and negative controls (Medium GC SUplex #2) were purchased from Invitrogen. Transfection with siRNA was carried out using Lipofectamine RNAiMAX (Invitrogen) according to the manufacturer's instructions.

### RNA isolation, cDNA synthesis, and quantitative real-time PCR

RNA was isolated using ISOGEN (Nippon Gene). First-strand cDNA was synthesized with reverse transcriptase (Superscript III, Invitrogen) and random primers in 20 µl of the reaction buffer. Synthesized first-strand cDNA was amplified by quantitative real-time PCR performed on an ABI Prism 7500 Sequence Detection System (Applied Biosystems), with qPCR™ Mastermix Plus for SYBR Green I (Eurogentech) for T-bet and RORγt, and qPCR™ Mastermix Plus (Eurogentech) for hypoxanthine-guanine phosphoribosyltransferase (HPRT) and 0.4 µM gene-specific primers. The primer pairs for T-bet and HPRT used in the quantitative real-time PCR were described before [5]. The primer pair for RORγt was: sense primer, 5′-TAC CTT GGC CAA AAC AGA GG-3′; anti sense primer, 5′-GAT GCC TGG TTT CCT CAA AA-3′.

### Statistical analysis

The Mann-Whitney U-test was performed for two-group comparisons. Dunnett's (one-way ANOVA) or Steel's (Kruskal Wallis test) test was performed for multiple group comparisons against a reference group. P values<0.05 were considered statistically significant. Error bars in all figures indicate standard errors of mean (SEM).

## Results

### The expressions of calpastatin and calpain are differently regulated during Th1, Th2, and Th17 development

**Figure 1:**
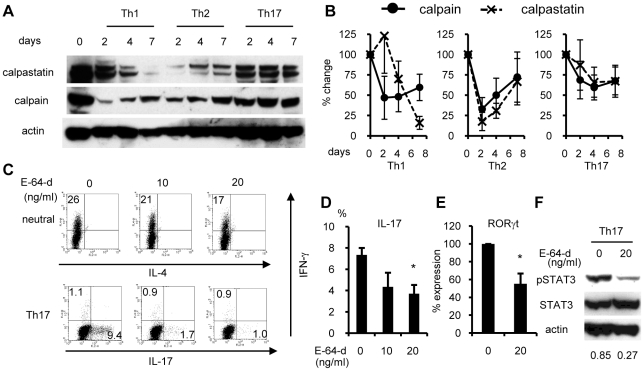
Calpastatin and calpain expressions and the effect of E-64-d on Th cell development. (A) Naïve CD4^+^ T cells were cultured under Th1-, Th2-, or Th17-promoting conditions, and the lysates were subjected to western blot analysis with the indicated antibodies. Data are representative of four independent experiments. (B) Band densities from the four independent experiments described in panel A were measured and normalized against the density of actin, and are depicted as the percent change from the value on day 0. (C) Naïve CD4^+^ T cells were maintained with the indicated concentrations of E-64-d under neutral or Th17-promoting conditions. On day 4 (Th17 condition) or day 6 (neutral condition), the cells were restimulated with PMA/ionomycin for 4 hours and subjected to ICS. Similar results were obtained in four independent experiments. (D) Statistical analysis of the IL-17 positive rates in panel C under Th17 conditions. *p<0.05 vs. absence of E-64-d (DMSO only, n = 4). (E) Naïve CD4^+^ T cells were maintained with the indicated concentrations of E-64-d under Th17-inducing conditions. On day 3, total RNA was extracted and subjected to quantitative RT-PCR analysis for RORγt. Expression levels were normalized to HPRT. Data depict the average+SE of the percent change from the value in the absence of E-64-d (DMSO only, n = 6). *p<0.05 vs. absence of E-64-d. (F) Th17 cells were cultured with the indicated concentrations of E-64-d. On day 3, the cells were washed and maintained with X-VIVO™ 20 (without FCS), and then lysed and subjected to western blot using the indicated antibodies on day 4. Similar results were obtained in four independent experiments. Numbers below the blots are the average relative density of pSTAT3 to actin.

To investigate the roles of calpain and calpastatin in Th cell development, we first examined their expressions over time during Th1, Th2, and Th17 development. Anti-CD3/28 monoclonal antibodies-stimulated naïve CD4^+^ T cells were cultured under neutral, Th1-, Th2-, or Th17-promoting conditions, and the lysates were subjected to western blot analysis. As shown in figure 1A–1B, the expression level of calpastatin consistently declined during Th1 development, and the calpain/calpastatin ratio in these cells eventually reached its maximum level for all the Th conditions. In contrast, the expression level of calpastatin gradually recovered during Th2 development after a transient decrease on day 2. The kinetics of calpain expression during the Th1, Th2, and Th17 developments were similar, but the degree of the transient decline was only modest in the Th17 conditions. Developing Th17 cells showed the smallest changes in calpain and calpastatin expression levels. In addition, intermediate trends of these proteins were observed in neutral conditions (Figure S1). These data suggested that the expressions of calpastatin and calpain were differently regulated during Th1, Th2, and Th17 development, and might modify the fate of Th development.

### E-64-d selectively inhibits Th17 development

We next studied the effect of E-64-d, a membrane-permeable calpain inhibitor, on the cytokine production in Th cells, and found that it had an effect in Th17 cells. Naïve CD4^+^ T cells were cultured with or without E-64-d under neutral or Th17 conditions, and restimulated with PMA/ionomycin for intracellular cytokine staining. E-64-d significantly suppressed the emergence of IL-17-positive cells under Th17 conditions in a dose-dependent manner (Figure 1C–1D). This suppression was associated with reductions in RORγt expression and STAT3 phosphorylation (Figure 1E–1F). Although some suppression of IFN-γ production by E-64-d was also observed under neutral conditions, it was modest and not evident under Th17 conditions (Figure 1C). These data suggested that the calpain inhibitor selectively suppresses Th17 development.

### Retroviral overexpression of calpastatin minimum functional domains

**Figure 2:**
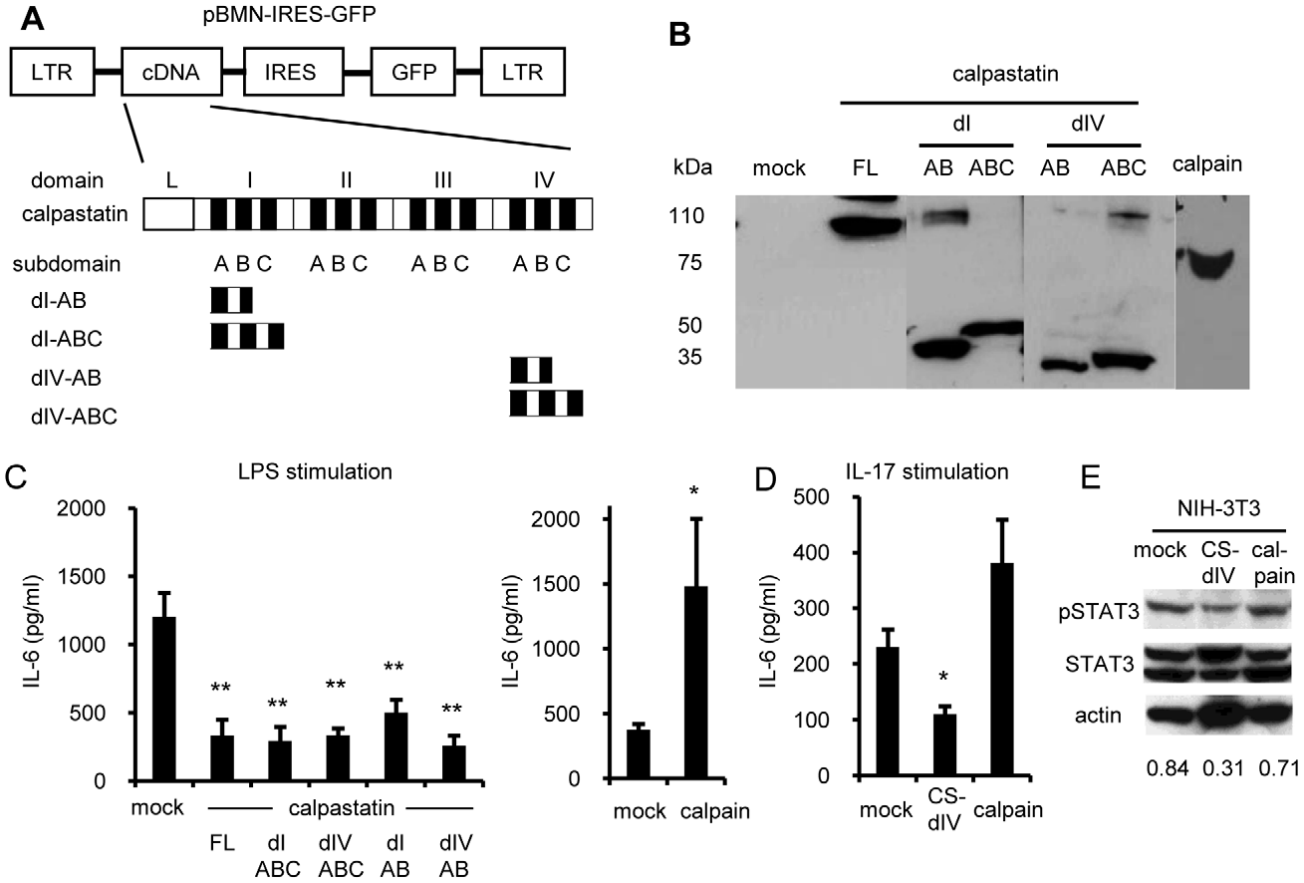
Retroviral expressions of calpastatin, calpastatin domains, and calpain and effect on IL-6 production from fibroblasts. (A) Retroviral construction and scheme of the domain structure of calpastatin. Domains I and IV are abbreviated as dI and dIV, respectively. (B) Calpastatin- or calpain-GRV was transfected into phoenix 293T cells by the calcium phosphate method. Three days later, the lysates of these cells were subjected to western blot analysis. The blots were probed with anti-calpastatin and anti-calpain antibodies. (C–D) Control cells, or calpastatin- or calpain-overexpressing NIH-3T3 cells (5x10^5^ cells/ml) were cultured with 1 µg/ml LPS (C) or 10 ng/ml IL-17 (D) for 24 hours. The culture supernatants were collected and IL-6 concentrations were measured by ELISA. Left panel of (C) shows mock vs. calpastatins (Dunnett's test, n = 5), and right panel shows mock vs. calpain (Steel's test, n = 5). Panel D shows the results of three independent experiments (Steel's test). *P<0.05 and **p<0.01 vs. mock. FL, full-length. (E) Control cells, calpastatin-, and calpain-overexpressing NIH-3T3 cells (infection efficiencies were >95%) were maintained without stimulation, then lysed and subjected to western blot analysis with the indicated antibodies. Similar results were obtained in three independent experiments. Numbers below the blots are the average relative densities of pSTAT3 to actin.

We found that the expression of calpastatin and calpain was differently regulated during Th1, Th2, or Th17 development, and that E-64-d suppressed Th17 development. However, since E-64-d inhibits not only calpain but also cathepsin [35], [36], it was possible that E-64-d's effect on Th17 development was a result of cathepsin blockage. Therefore, we constructed a retrovirus system to overexpress calpastatin, the specific inhibitor of calpain. As shown in figure 2A, we generated mock-, calpastatin-, and calpain-integrated retroviruses with a GFP reporter (mock-, calpastatin-, and calpain-GRV). Anti-CD3/28 monoclonal antibodies stimulated naïve CD4^+^ T cells were cultured under neutral conditions and infected with these retroviruses. The efficiencies of infection were determined from the GFP expression using flow cytometry. However, compared with the mock-GRV-infected cells, the expression of full-length (FL) calpastatin-GRV in infected CD4^+^ T cells was not maintained, and the positive cells had almost disappeared by day 7 after infection (Figure S2). As this might have been owing to the length and/or sequence of the calpastatin cDNA, we next cloned minimum functional domains of calpastatin by PCR.

As shown in figure 2A, the calpastatin cDNA contains four homologous functional domains (I to IV), and domains I and IV have a higher calpain-inhibiting activity than domains II and III [37]. Each inhibitory domain has three sub-domains A, B, and C: sub-domains A and C bind to calpain, and subdomain B is crucial for calpastatin's inhibitory activity. Therefore, we cloned domains I and IV, as just subdomains A and B (CS-dIAB, CS-dIVAB) or all three subdomains (CS-dIABC, CS-dIVABC). These retrovirally overexpressed minimum domains of calpastatin showed much better stability in infected cells than the full-length sequence (Figure S2).

### Overexpression of calpastatin minimum functional domains suppresses the IL-6 production from fibroblasts

To confirm the overexpression of calpastatin and calpain proteins, we transfected the retroviruses into Phoenix 293T cells, and then subjected the lysates of these cells to western blot analysis. We detected the expected sizes of calpastatins and calpain proteins (Figure 2B). In addition, to check the function of the minimum functional domains of calpastatin with different sets of subdomains and calpain at the protein level, a casein-based calpain activity assay was performed using lysates from calpastatin- or calpain-overexpressing NIH-3T3 cells, as described previously [19], and the calpain inhibitor function was confirmed for all these constructs (data not shown).

As we previously found that E-64-d suppresses IL-6 production in a fibroblast-like synovial cell line [33], we examined the biological function of the calpastatins and calpain by the IL-6 production using NIH-3T3 cells. Control cells and calpastatin- or calpain-overexpressing NIH-3T3 cells (infection efficiencies were >95%) were stimulated with LPS (Figure 2C) or IL-17 (Figure 2D), and the IL-6 concentration in the culture supernatants was measured by ELISA. The IL-6 induction was significantly suppressed in cells overexpressing all forms of calpastatin similarly, whereas the calpain-overexpressing cells produced more IL-6 than mock-infected cells (Figure 2C–2D). These results suggested that the target molecule of calpain and calpastatin might lie in the common signal pathway of IL-6 gene expression such as the NF-κB signal rather than in the receptor level. In addition, in the absence of LPS stimulation, the constitutive phosphorylation of STAT3, which is an important effector of IL-6 signal transduction, was also suppressed in calpastatin-overexpressing NIH-3T3 cells as a result of the IL-6 suppression (Figure 2E). On the other hand, no upregulation of phosphorylated STAT3 was observed in calpain-overexpressing NIH-3T3 cells, probably because of the absence of LPS stimulation.

Because of its stability and strong biological effect, we used the dIV-ABC construct of calpastatin in further experiments (designated just as CS-dIV).

### Production of IL-17 and IL-6 was suppressed in calpastatin-overexpressing CD4^+^ T cells

**Figure 3:**
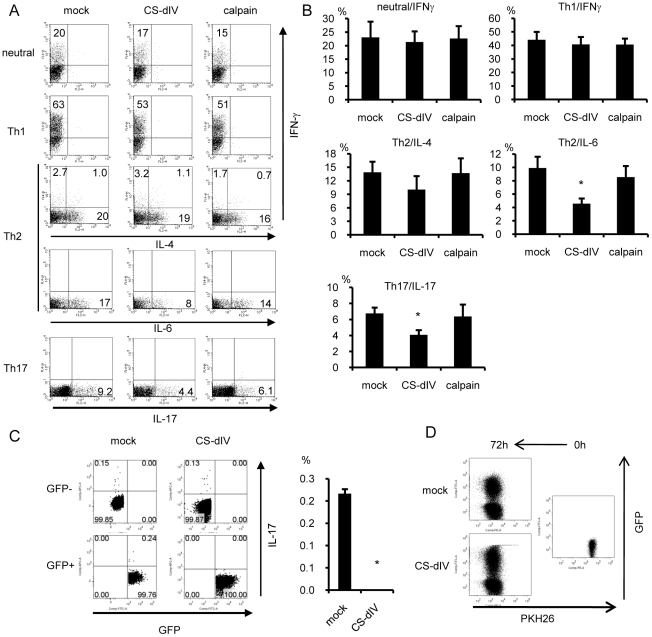
Calpastatin-overexpression in Th cells resulted in decreased IL-6 and IL-17 production. (A) Naïve CD4^+^ T cells were cultured under neutral, Th1, Th2, or Th17 conditions. On day 1, these cells were infected with mock-GRV or calpain- or CS-dIV-GRV and expanded on day 2 with IL-2. On day 4 (Th17 conditions) or on day 6 (neutral, Th1, and Th2 conditions), the cells were restimulated with PMA/ionomycin for ICS. Representative results for GFP^+^-gated cells are shown (n = 11 for IFNγ, n = 10 for IL-4, n = 6 for IL-6 and IL-17). (B) Statistical analysis of the results in panel A by Steel's test. (C) Naïve CD4^+^ T cells were cultured under neutral conditions and infected with mock-GRV or CS-dIV-GRV. On day 6, these cells were restimulated with PMA/ionomycin for FACS. Representative data of GFP^+^, GFP^−^-gated cells are shown in the left panel, and the statistical analysis is shown in the right panel (n = 3) (D) Retrovirus-infected Th cells were stained with PKH-26 dye and then stimulated with anti-CD3/28 monoclonal antibodies. The fluorescence intensity of the PKH-26 dye in infected (GFP^+^) and non-infected (GFP^−^) cells was analyzed by FACS. *p<0.05 vs. mock.

We next investigated the effect of calpastatin and calpain overexpression on cytokine production in Th cells. Calpastatin- or calpain-overexpressing CD4^+^ T cells were cultured under neutral, Th1-, Th2-, or Th17-promoting conditions, and cytokine production was analyzed by intracellular cytokine staining. Compared with mock-infected or calpain-overexpressing Th cells, those overexpressing CS-dIV contained fewer IL-6- and IL-17-positive cells under Th2 and Th17 conditions, respectively (Figure 3A–3B). Furthermore, CS-dIV overexpression completely blocked the emergence of IL-17-positive Th cells under neutral conditions (Figure 3C). On the other hand, calpain-overexpressing CD4^+^ T cells showed little change with regard to the production of IFN-γ and IL-4. We also checked the effect of CS-dIV overexpression on cell proliferation using PKH26 labeling together with GFP expression, and found no difference in the cell-division rates between mock- and CS-dIV-infected cells (Figure 3D). These data suggested that the overexpression of CS-dIV suppressed IL-17 and IL-6 production without causing cytotoxic effects.

**Figure 4:**
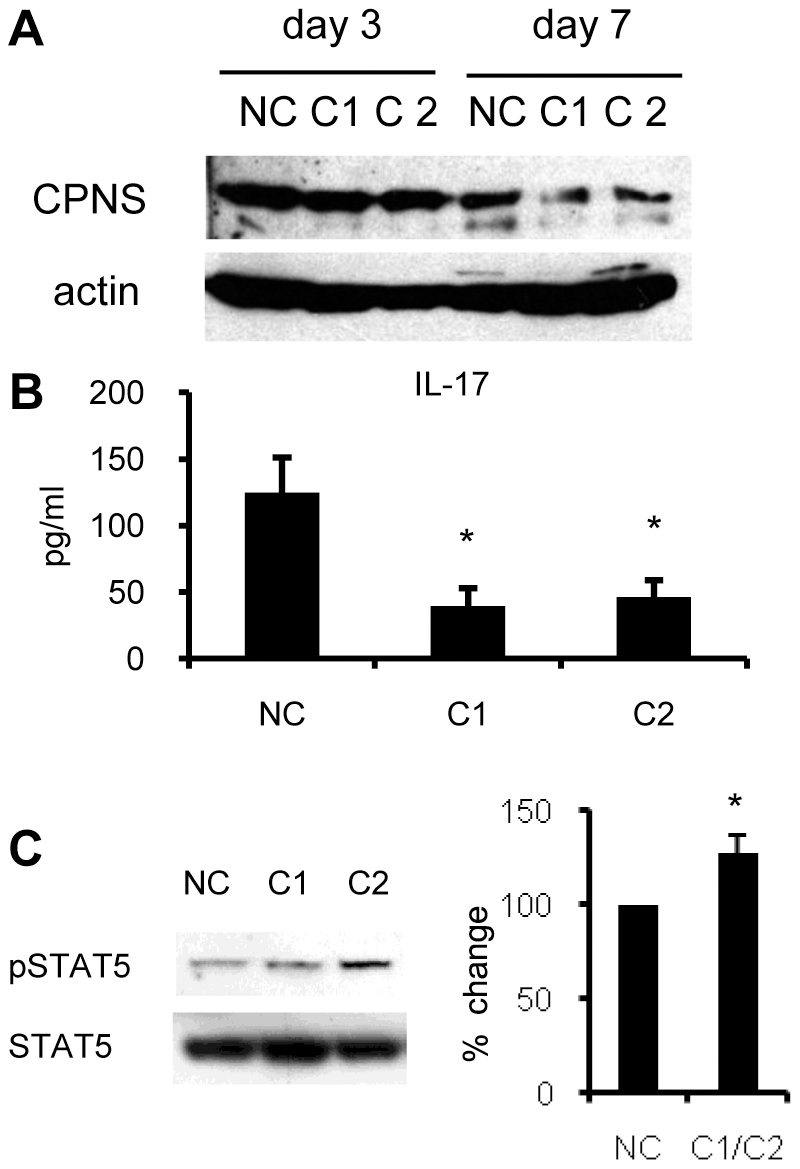
Silencing of the calpain small subunit suppressed the IL-17 production from Th cells. (A) Naïve CD4^+^ T cells were cultured under neutral conditions, and treated with siRNA against calpain small subunit (CPNS). This experiment was carried out using two siRNAs (CPNS1; C1 and CPNS2; C2). On day 3 and 7, these cells were lysed and subjected to western blot analysis. NC; negative control. (B) On day 6, the cells in panel A were washed 3 times and restimulated with an anti-CD3 antibody for 48 hours. The culture supernatants were then collected, and IL-17 was measured by ELISA. Statistical analysis of eight independent experiments is shown (Steel's test). (C) On day 6, the cells in panel A were incubated with X-VIVO™ 20 (without FCS) for the last 6 hours, then lysed and subjected to western blot analysis with the indicated antibodies. Similar results were obtained in three independent experiments. The band densities of pSTAT5 were measured and normalized to that of total STAT5, then the percent change from NC was calculated. Statistical analysis of three independent experiments is shown in the right panel. *p<0.05 vs. NC.

To confirm the suppression of IL-17 production by calpastatin, we silenced the expression of the small subunit of calpain (CPNS), a regulatory subunit of calpain, in CD4^+^ T cells using an RNAi approach. The knockdown of CPNS is known to reduce the large subunit of both calpain I and II at the protein level and to interfere with calpain function [38], [39]. We achieved a significant decrease, but not complete depletion, of protein on day 7 after treatment with the siRNA for CPNS probably because of the relatively long half-life of calpain at the protein level (Figure 4A). We then analyzed the IL-17 production under neutral conditions by ELISA on day 7, and confirmed that the IL-17 production was suppressed in Th cells treated with siRNA against CPNS (Figure 4B). We supposed that calpain inhibition suppressed IL-17 production by up-regulating the STAT5 signal via stabilization of the common cytokine receptor gamma (γ_c_) chain [40], because cytokine signaling through the γ_c_ chain and STAT5 is critical for Th17 generation [41], [42]. As shown in figure 4C, we confirmed that the constitutive phosphorylation of STAT5 was up-regulated in the CPNS-siRNA treated T cells, and not in control cells.

### Overexpression of the minimal functional domain of calpastatin suppresses NF-κB signaling by stabilizing IκBα

**Figure 5:**
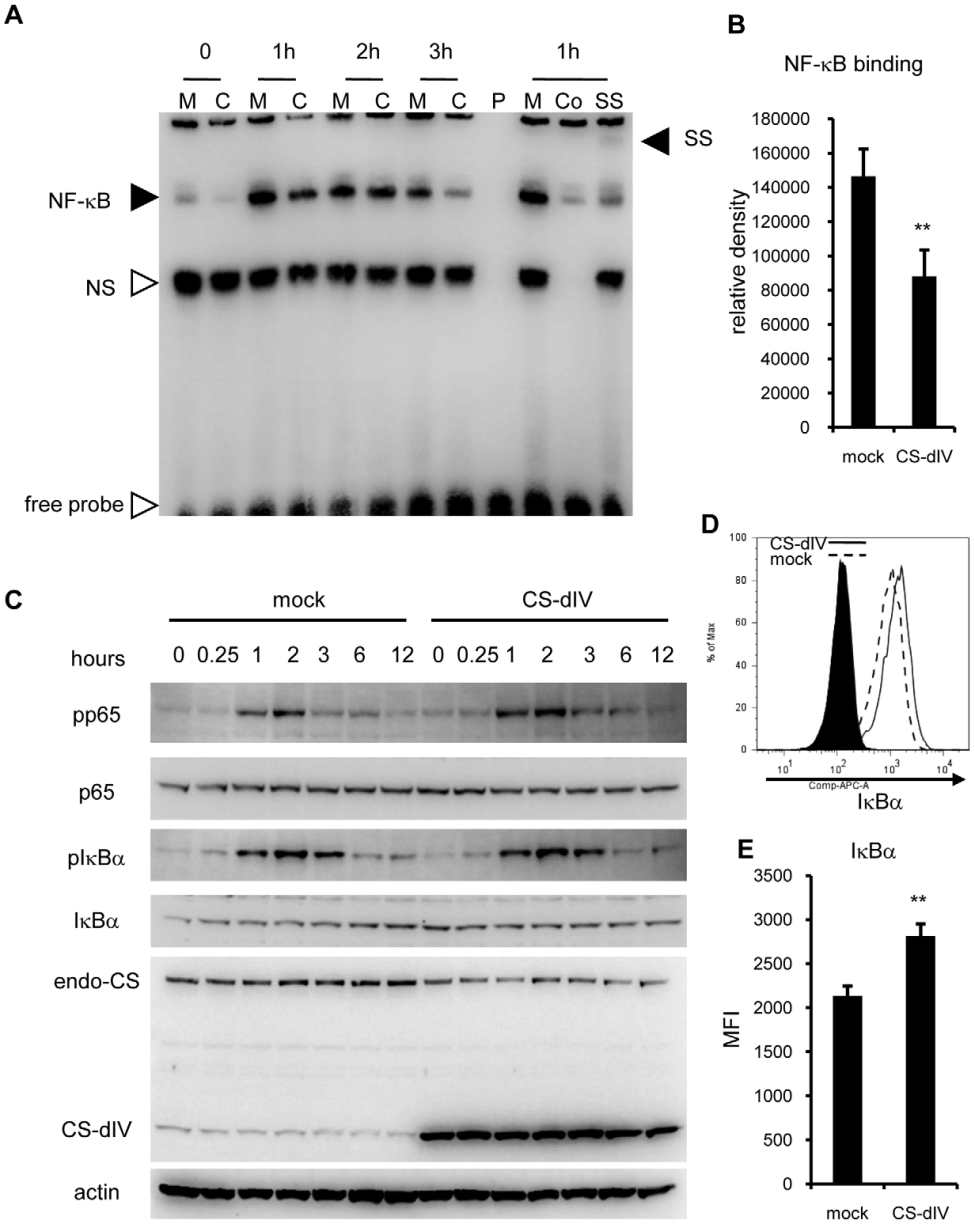
Overexpression of the minimal functional domain of calpastatin suppressed NF-κB signaling via stabilization of IκBα. (A) Nuclear extracts from mock (M)- and CS-dIV (C)-overexpressing NIH-3T3 cells stimulated with LPS were subjected to EMSA. P, probe only; Co, competition by preincubation with non-labeled probe; SS, super shift by preincubation with an anti-p65 antibody; NS, non-specific bands. Similar results were obtained in six independent experiments. (B) Bands densities of NF-κB were measured and statistical analysis was performed. (C) Cytoplasmic extracts from the same cells used in panel A were subjected to western blotting with the indicated antibodies. Similar results were obtained in three independent experiments. (D) Mock- or CS-dIV-overexpressing NIH-3T3 cells were stained with IκBα antibodies intracellularly and analyzed by flow cytometry. Similar results were obtained in six independent experiments. (E) Statistical analysis of the MFI of IκBα shown in panel C. **p<0.01 vs. mock. endo-CS; endogenous calpastatin.

The molecular mechanism by which calpain inhibitors suppress IL-6 production is reported to be the suppression of NF-κB signaling by blocking calpain's degradation of IκB [39], [43], [44], [45]. However, it was not known whether the calpastatin minimum functional domains have the same function as full-length calpastatin. To test this mechanism, we first analyzed the NF-κB signaling by electrophoresis mobility shift assay (EMSA) using nuclear extracts from mock- and CS-dIV-overexpressing NIH-3T3 cells. As shown in figure 5A, the CS-dIV-overexpressing NIH-3T3 cells showed not only a suppressed peak of NF-κB binding to the target sequence, but also a shorter duration of NF-κB binding induced by LPS stimulation. We also confirmed that the bands of NF-κB were specifically p65 by super shift assay. The mean percent reduction in NF-κB binding at 1 hour after LPS stimulation by CS-dIV overexpression was 58%, which was statistically significant (Figure 5B). We next examined the upstream signaling of NF-κB by western blot using cytoplasmic extracts of the same cells. As shown in figure 5C, we confirmed that there were no significant changes in upstream NF-κB signaling events (the phosphorylation of p65 and IκBα) between the mock- and CS-dIV-overexpressing NIH-3T3 cells.

We also observed an increase in the total IκBα protein in CS-dIV-overexpressing NIH-3T3 cells at steady state (0 hours) and up to 1 hour after LPS stimulation. To confirm the increase in total IκBα protein in the CS-dIV-overexpressing NIH-3T3 cells more precisely, we performed intracellular staining for IκBα, and determined the mean fluorescence intensity (MFI) by flow cytometry (Figure 5D–5E). We observed a significant increase in IκBα-MFI (30%) in the CS-dIV-overexpressing NIH-3T3 cells. These data confirmed that the overexpression of CS-dIV resulted in increased IκBα and decreased NF-κB binding to its target DNA in the nucleus, without decreasing their upstream signaling.

### Overexpression of the minimal functional domain of calpastatin suppresses IL-6 production in synovial fibroblasts derived from RA patients

**Figure 6:**
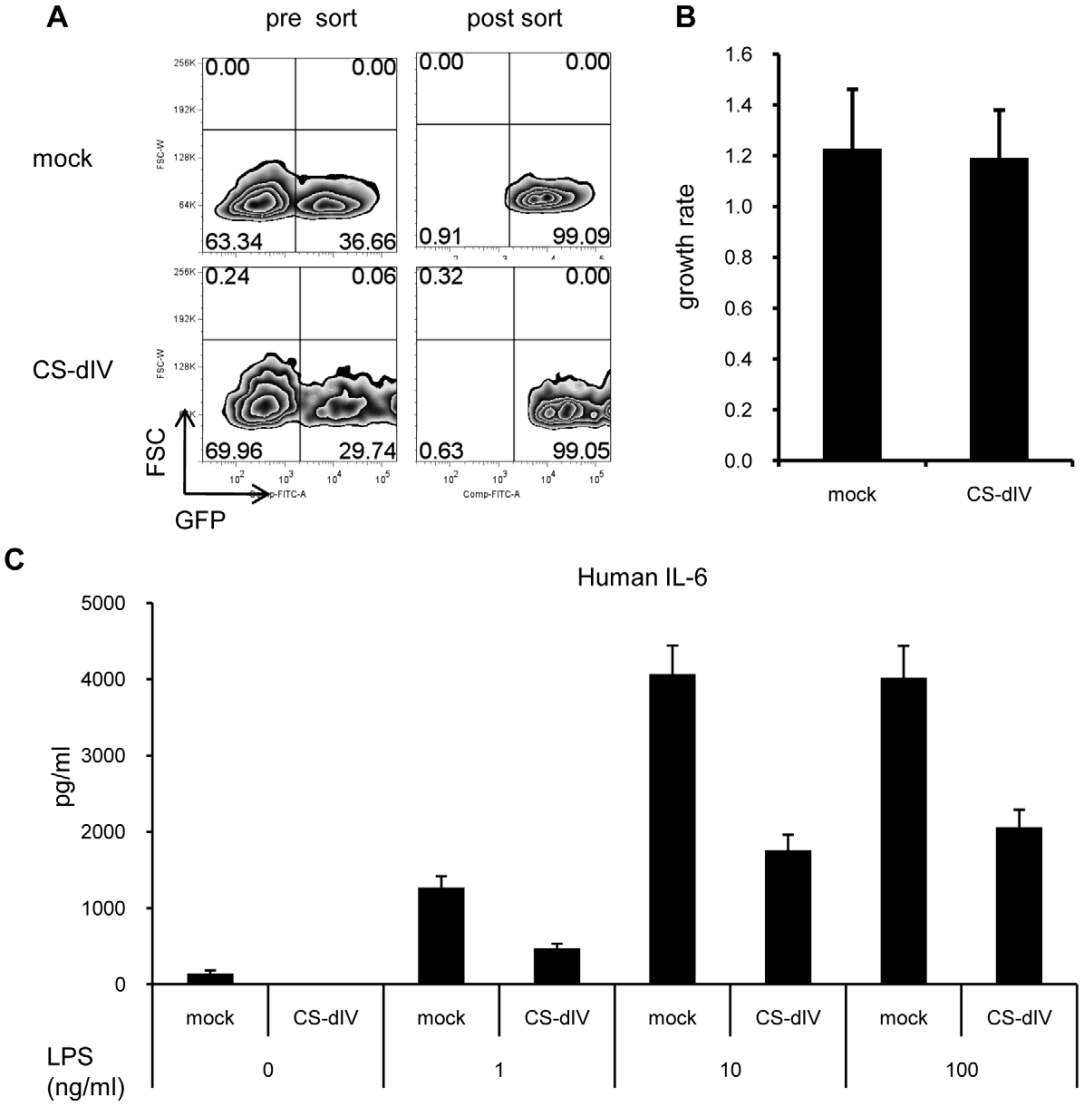
Calpastatin overexpression in the synovial fibroblasts of RA patients efficiently suppressed IL-6 production. (A) Synovial fibroblasts derived from RA patients were subjected to mock- or CS-dIV-GRV infection, and the infected cells were sorted by FACS. Sorting purity was >99%. (B) The sorted GFP^+^ cells in panel A were seeded into 96-well plates at 1000 cells/well. Forty-eight and 72 hours after seeding, ATP was measured using a CellTiter-Glo® Assay kit. Summarized data from three independent experiments are shown. (C) The cells used in panel B were stimulated with the indicated concentration of LPS for 24 hours, and IL-6 in the culture supernatant was measured by ELISA. Similar results were obtained in three independent experiments.

To confirm the anti-inflammatory effect of CS-dIV on primary fibroblasts, we isolated synovial fibroblasts from RA patients and infected these cells with mock- or CS-dIV-expressing retrovirus. The infected cells were then sorted according to GFP expression using FACS (purity >99%) (Figure 6A). These sorted cells were then stimulated with LPS, and the IL-6 in the supernatant was measured by ELISA. As shown in figure 6C, the overexpression of CS-dIV suppressed not only the LPS-induced IL-6 production, but also the basal production, completely. In addition, there was no difference in the cell proliferation between the mock- and CS-dIV-overexpressing synovial fibroblasts (Figure 6B). All of these data suggested that the inhibition of calpain by a minimal functional domain of calpastatin elicits a coordinated anti-inflammatory effect by the inhibition of both IL-6 and IL-17 from not only T cells but also synovial fibroblasts.

## Discussion

Calpain is known to be present in the synovial fluid of RA patients [27], [29], [46] and in the arthritic joints of CIA mice [28]. Because calpain has proteoglycanase activity [29], [47], it causes cartilage damage in the joints of RA patients [28], [46], and is concomitantly involved in osteoclastic bone resorption [48]. In addition, autoantibodies against calpastatin, a natural and specific inhibitor of calpain, are widely observed in RA and psoriasis patients [19], [20], [21], and positivity for anti-calpastatin antibodies is known to correlate with serological markers of the disease activity [23], [24]. These findings strongly suggest that an insufficiency of calpastatin or dominance of calpain contributes to the pathogenesis of inflammatory processes such as RA. In fact, we previously reported that the administration of E-64-d ameliorates the arthritis in CAIA mice via decreased IL-1β and IL-6 production in synovial cells [33]. However, E-64-d is not a specific inhibitor of calpain; it also inhibits cathepsins. Therefore, a more specific experimental system for inhibiting calpain function was required to clarify its effect on inflammatory cells. Moreover, little is known about the effect of the calpain-calpastatin balance on Th cells.

In the present study, we established a system for overexpressing calpastatin subdomains in primary cells and in cell lines, using a retroviral expression system, and a CPNS knock-down system using siRNA to achieve the specific loss of calpain function. Notably, we generated a minimal functional domain of calpastatin (CS-dIV) that was stably expressed in both cell lines and primary cells, even though full-length calpastatin only showed short-term expression. There might be some sequences in the full-length calpastatin cDNA that are incompatible with the retrovirus vector, or its length might simply be the problem. It is also possible that the overexpression of full-length calpastatin has some toxic effects on cells, while the minimal functional domain of calpastatin does not. In fact, no toxicity was observed when CS-dIV was overexpressed in either primary Th cells or fibroblasts. In any case, these properties of CS-dIV were advantages for its use in analyzing the gain of calpastatin function in vitro.

Using CS-dIV overexpression to obtain the specific loss of calpain function in vitro, we demonstrated that it suppressed IL-6 production by reducing NF-κB signaling via the inhibition of IκBα degradation, without affecting the upstream signals of NF-κB, in NIH-3T3 cells. Several reports have shown that IκB is a substrate of calpain, and anti-calpain compounds block IκB's degradation, consequently reducing NF-κB signaling [44], [45], [49], [50], [51]. Because all of these reports utilized calpain inhibitors that are not specific for it, the results may include effects caused by inhibiting molecules other than calpain. Although two other reports showed similar findings using a calpastatin overexpression system, these were performed in cell lines [39], [43]. In the present study, we confirmed that the overexpression of CS-dIV suppressed IL-6 production by not only NIH-3T3 cells but also primary Th cells and fibroblasts derived from the joints of RA patients. IL-6 is recognized as a major proinflammatory cytokine responsible for the pathophysiology of inflammatory diseases such as RA; the IL-6 level in synovial fluids correlates with disease activity [52]. Moreover, an anti-IL-6 receptor antibody suppresses RA in human patients [53], [54], [55]. Notably, we found that the overexpression of CS-dIV completely blocked the constitutive production of IL-6 from inflammatory fibroblasts derived from RA patients. This result supports the clinical application of directly overexpressing CS-dIV in inflammatory sites such as the joints of RA patients.

NF-κB signaling is essential for IL-6 production, and IL-6 plays a crucial role in IL-17 development [11]. Furthermore, NF-κB is known to be activated in the synovium of patients with RA [56], [57], [58]. Therefore, the inhibition NF-κB signaling is a promising strategy for controlling inflammatory diseases. However, although many inhibitors to NF-κB are aggressively under development, their specificity and toxicity are common problems [59]. In the present study, we observed that only a moderate increase in IκBα (+30%) had a relatively large impact on the suppression of NF-κB signaling (−58%) in NIH-3T3 cells. This finding suggests that strategies such as increasing IκBα or protecting it from degradation by calpain may efficiently inhibit NF-κB. In fact, our results of CS-dIV overexpression in primary fibroblasts derived from the joints of RA patients showed that CS-dIV had a strong suppressive effect on the spontaneous production of IL-6.

We next demonstrated that the inhibition of calpain activity by overexpressing a minimal functional domain of calpastatin or by CPNS-siRNA suppressed not only IL-6 but also IL-17 production by Th cells. We supposed this effect resulted from the inhibition of Th17 development via upregulation of the STAT5 signal, rather than from the direct suppression of IL-17 expression. This scenario is consistent with previous reports [40], [41], [42]. We also tested the effect of calpain inhibition by CPNS-siRNA on other STAT signals (STAT1, STAT4, and STAT6), and observed no obvious changes (data not shown).

These data suggested that the calpain-calpastatin balance specifically moderates STAT5 signaling, and suppresses Th17 development preferentially. Although Th17 cells are believed to assume significant roles in inflammatory disease, controversy remains over whether this is true in human RA [12], [13], [60]. To address this issue, we and another group reported that the synovial tissues and fluids of RA patients contain predominantly IFN-γ producing CD4^+^ T cells, and that the emergence of Th17 cells is neither increased in RA nor correlated with disease activity [14], [15]. However, more recently, Leipe et al., using samples from very early active and treatment-naïve RA patients, reported that the percentage of Th17 cells correlates strongly with the activity of RA, and concluded that Th17 cells play an important role in human RA [61]. Furthermore, anti–IL-17A antibodies (such as AIN457) are currently being investigated for the clinical treatment of autoimmune diseases, including RA, psoriasis, and Crohn's disease, and therapeutic effects are starting to be reported [62]. Therefore, the suppression of both Th17 development and IL-6 production by T cells and non-T cells, through overexpressing the calpastatin subdomain could be a sophisticated strategy for treating inflammatory diseases such as RA.

In conclusion, the inhibition of calpain function by calpastatin overexpression suppresses Th17 development via an upregulation of STAT5 signaling, and reduces IL-6 production via a repression of NF-κB signaling in both T cells and non-T cells (for example, synovial fibroblasts and macrophages). Not only does calpain have immunological functions, but it also degrades the matrix component of articular cartilage; therefore, the blockade of calpain function could be a promising strategy for treating inflammatory diseases such as RA. Because CS-dIV is a relatively small polypeptide, coupling it with cell penetrating peptide (CPP) will not be difficult. If CPP-coupled CS-dIV can be synthesized efficiently, it will be a novel candidate for the therapeutic control of RA-associated joint inflammation, because it can be injected locally and will penetrate inflammatory cells, in which it will function as both an NF-κB and an IL-17 inhibitor, without the risk of systemic effects.

## Supporting Information

Figure S1 **The expressions of calpastatin and calpain under neutral conditions in Th cells over time.** Naïve CD4^+^ T cells were cultured under neutral conditions, and the lysates were subjected to western blot analysis with the indicated antibodies. Data are representative of three independent experiments.
(TIF)

Figure S2 **Retrovirally transfected proteins in naïve T cells over time.** Naïve CD4^+^ T cells obtained from the splenocytes of BALB/c mice were infected with mock-, calpastatin-, calpain-, or modified calpastatin-GRV, and the infection efficiency was determined from the GFP expression using flow cytometry. Data are representative of seven independent experiments.
(TIF)
