# Supplementary figures and images for: Overexpression of a Minimal Domain of Calpastatin Suppresses IL-6 Production and Th17 Development via Reduced NF-κB and Increased STAT5 Signals
Source: PLoS One. 2011 Oct 27;6(10):e27020. doi: 10.1371/journal.pone.0027020 (PMC3203168; doi:10.1371/journal.pone.0027020)

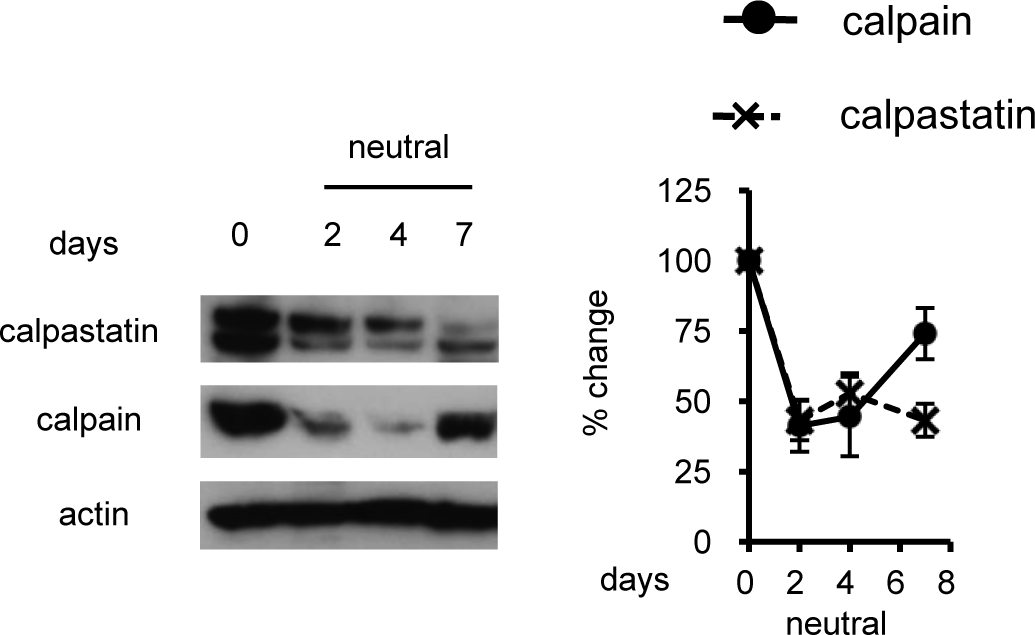

Supplement: Figure S1 — The expressions of calpastatin and calpain under neutral conditions in Th cells over time. Naïve CD4+ T cells were cultured under neutral conditions, and the lysates were subjected to western blot analysis with the indicated antibodies. Data are representative of three independent experiments. (TIF) [file pone.0027020.s001.tif]

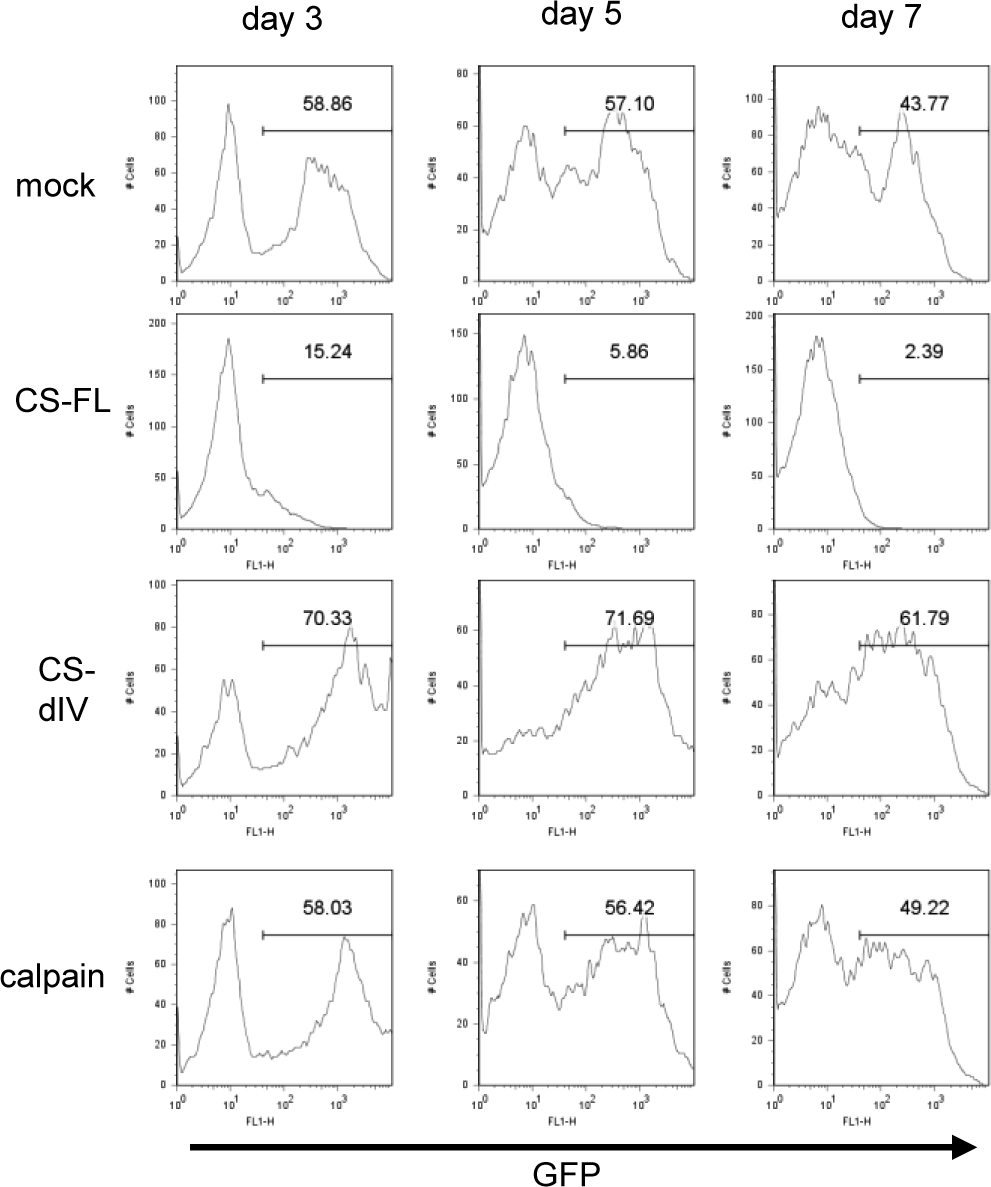

Supplement: Figure S2 — Retrovirally transfected proteins in naïve T cells over time. Naïve CD4+ T cells obtained from the splenocytes of BALB/c mice were infected with mock-, calpastatin-, calpain-, or modified calpastatin-GRV, and the infection efficiency was determined from the GFP expression using flow cytometry. Data are representative of seven independent experiments. (TIF) [file pone.0027020.s002.tif]
